# Daily life and life quality 3 years following prostate cancer treatment

**DOI:** 10.1186/1472-6955-12-11

**Published:** 2013-04-10

**Authors:** Liselotte Jakobsson, Lena Persson, Pia Lundqvist

**Affiliations:** 1 School of Health and Society, Kristianstad University, 291 88 Kristianstad, Sweden; 2 Division of Nursing, Department of Health Sciences, Lund University, Lund, Sweden

**Keywords:** Prostate cancer, Longitudinal case study, Quality of life, Sexual life, Sense of coherence, Interview, Mixed method

## Abstract

**Background:**

Knowledge of experiences from prostate cancer is sparse in a longitudinal perspective. From a nursing perspective, results from combined qualitative and quantitative studies are lacking however would present the broadest knowledge base for best practice. Present descriptions of medical-physical symptoms such as urinary, bowel and sexual dysfunction from quantitative inquiries need be complemented with qualitative results. Such knowledge is essential in relation to treatment and communication with patients over the years and not only shortly after surgery.

**Methods:**

A longitudinal study was formatted to investigate general and specific health quality and sense of coherence quantitative alterations over three years. A general health quality module (EORTC QLC-C30) and a disease-specific module (EORTC PR-25) were applied for the longitudinal study together with the Orientation to life questionnaire (SOC), measuring a persons’ sense of coherence. In order to strengthen reliability and compensate for low participation we used the Directed content analysis for interviewing and analysis. The method allows using findings from earlier research when interviewing along with detecting new areas. Twenty-one men were followed over three years and six of them, in the third year, accepted to be interviewed.

**Results:**

We found high quality of life ratings and extended the study with follow-up interviews in year three, to investigate whether questionnaire results were in line with interview findings. We found high life quality and functioning ratings that were in line with qualitative descriptions. Interview analysis showed retrieval of life as lived before, yet in a different way, the men never forgot the diagnosis event, had a unique illness history worth hearing, and had come to terms with most treatment-related shortcomings. Sense of coherence ratings were medium to high and confirmed stability over time in comprehensibility, manageability and meaningfulness after prostate cancer treatment.

**Conclusions:**

Over the years, the men’s negative experiences from shifted into ‘a good life’ though in a different way than before. The interpretation is supported in the study by quantitative results showing a high degree of functioning. The men’s sense of coherence seamed to support their handling of life three years after prostate cancer treatment.

## Background

How life is perceived by men treated for prostate cancer years after surgery is little known from a Swedish perspective. There are extensive descriptions of medical-physical symptoms such as urinary, bowel and sexual dysfunction from quantitative inquiries [1]. Less is described from a qualitative perspective based on the men’s descriptions. From a nursing perspective, results from combined method studies describing both quantity and quality aspects on prostate cancer sequels are lacking, though they would be useful as they contain a wide area of information and constitute a solid ground for knowledge. Such knowledge is essential in relation to treatment and communication with patients over the years and not only shortly after surgery.

In Sweden the average annual increase of prostate cancer over the last 10 years is 0.2%. The introduction of PSA testing explains the large increase in the late 90s and early 2000s. The incidence has decreased during the last few years and there were 10 317 new cases reported in 2009 [2]. Because the illness is common, men with prostate cancer are often met in both in- and out-patient nursing care settings.

Men’s perception of the impact of the illness, and treatment side effects, is related to the individual man’s ability to handle life issues in general. The more life is perceived to have meaning, the better the chances are of handling a life-threatening event such as a cancer diagnosis. An often applied theoretical foundation for analysing cancer care experiences is the salutogenic concept developed by Antonovsky. Antonovsky proposed the salutogenic concept to explain why people stay healthy during extreme life-changing conditions. Antonovsky developed the Orientation to life questionnaire, referred to as the Sense of Coherence scale, SOC, to measure people’s ability to handle different life situations [3]. The SOC scale measures a person’s ability to perceive comprehensibility, manageability and meaningfulness in life and is considered to be a person-related characteristic that also influences the person’s ability to cope [4]. In an article from northern Sweden of men and women (n = 837 n = 882, respectively) with stomach trouble, identified illnesses (stroke, cardiac infarction, diabetes, anti-hypertension treatment) and a disease-free control group, a correlation was found between low SOC scores and poor perception of health, low social support and low emotional support [5]. Whether men treated for prostate cancer rate their sense of coherence high and weather there is a correlation to life quality is sparsely known.

When measured as a quantitative dimension, health-related quality of life is often used in prostate cancer care as an outcome of cancer illness *per se*, as assessment of treatment for various levels of malignancy and in connection with a variety of aspects [6,7]. In prostate cancer care, generic instruments for assessment of health-related life quality are often applied, together with disease-specific questionnaires such as the EORTC Quality of life Questionnaire [8] and the QLQ PR25 [9], measuring prostate cancer-specific questions on urination, stomach and sexual impairment. Outcomes of such quantitative studies give a structured and rigorous picture of how life is perceived during and after treatment, and of treatment side effects at a group level. Measurement results are often compiled from a large number of participants [10]. We agree with Gacci *et al.* that result from this and other studies contribute to the clinicians’ ability to counsel long-term prostate cancer survivors. In a quantitative study by Johansson *et al.* (n = 481), men with prostate, men and women with gastrointestinal cancer and, women with breast cancer rated their quality of life comparable to a general sample. A small difference was seen in the investigation group compared to the ‘normal’ population control group at baseline and the difference levelled out in three months’ time [11]. However, Jakobsson *et al.*, found lower levels of general life quality among men with prostate cancer (n = 155) compared to men in the general population [12]. Zenger also found lower ratings among men with prostate cancer compared to a general population sample (n = 265) [1]. If ratings fluctuate over time is however not presented in the studies. Fluctuation over time is found in the work of Taminiau-Bloem *et al.* who reported major changings in answering from baseline to follow up, in a sample of 50 cancer patients in aspects underlying OQL appraisal of *e.g.* taking a short walk, pain, fatigue, worry and overall quality of life [13]. Their finding calls for caution when giving attention to both quantitative and qualitative follow-up assessment. Quantitative studies complemented with a qualitative approach at an individual level, including side effects from treatment and general handling of illness sequels and health care actions, are lacking and this is a shortcoming for individual nursing and healthcare treatment. The aim of this study was to describe men’s experiences of life following prostate cancer from health-related quality of life and a salutogenic perspective and, to complement quantitative data with qualitative descriptions.

## Methods

### Sample

Men visiting a urological out-patient ward for surgery follow-up, were consequently asked to participate in the longitudinal study – 3 years.

### Methods

The Orientation to life questionnaire, SOC, with 13 items was used in the study. The scale assesses the three components of the sense of coherence concept; comprehensibility, manageability and meaningfulness. The scale’s response format ranges from 1 to 7 on each facet of the concept [14]. The European Organisation for Research and Treatment of Cancer, EORTC, Quality of Life, QLQ, C-30 questionnaire [8], combined with the module for prostate cancer, PR-25 [9] were applied annually throughout the three years. The QLQ C-30 contains 30 questions and was developed by the EORTC Quality of life study group. It explores functional areas such as physical, role, emotional, cognitive, and social functioning, as well as overall health and quality of life. It also includes multi-item scales and single items assessing fatigue, nausea and vomiting, pain, dyspnoea, insomnia, appetite loss, constipation and diarrhoea and, financial impact. All items but two scores from 1 = not at all to 4 = very much, while the overall health status/life quality questions scores from 1 = very poor to 7 = excellent. Finally, the prostate-specific module, PR-25, was applied. The questionnaire contains 25 questions and was developed by the EORTC Genito-Urinary Tract Cancer Cooperative Group [9]. The scale assesses symptoms related to sexual problems, urination, bowel and hormonal treatment, where higher scores represent more symptoms. In sexual functioning, higher scores represent higher levels of functioning. Each item scores from 1 = not at all to 4 = much.

Ratings of the Sense of Coherence were summarized, and a mean value for each person was used for statistical calculations. A low score represents lower perceived handling of life. Raw scores for the QLQ-C30 and the PR25 were transformed into a 100-point scale [15], where functional scales (0–100) with high ratings represents high functioning, except for scale of fatigue, pain and nausea and vomiting where low scores represent high functioning. For single item scores (0–100), lower scores represent better functioning, *i.e.* less symptoms and problems. According to clinical practice, differences of 5–10 scale points indicate a small change and differences of 10–20 points indicate a moderate change [16]. Differences between year one and two were assessed with Wilcoxon signed rank test for dependent group comparison, with a significance level at p = <0.05. Differences between all three years were assessed with Friedman 2-way ANOVA-test for dependent group comparison. Correlations between EORTC C-30, PR 25 and SOC were performed with Kendall’s Tau-b analysis (not in table). Non-parametric tests were mostly used because of a non normal distribution [17]. The SPSS software, 19.0, was used for all statistical analyses. A quantitative mail-out/mail-in questionnaire survey was performed with one reminder sent per year.

In the third year (2005) the study was extended, and the men were asked to participate in a qualitative follow-up interview. Interviews were performed in the men’s homes or workplaces with focus on how they perceived life after surgery. The interviews were recorded, lasted for 60–70 minutes, transcribed verbatim and analysed according to Hseih and Shannon’s directed content analysis [18]. The directed content analysis builds on earlier research findings and might support and extend an existing cluster of knowledge of a certain illness or condition. The model also focuses new aspects revealed in interviews. Our study focused life quality described from the men’s perspective, experiences on an emotional and physical level and commonly described shortcomings (sexual, urinary, bowel problems) [11,19,20]. Initially the three authors read through the texts independently and discussed the meaning of the texts as a whole to get a deep understanding of the men’s experiences. Each author then coded the texts in line with the predetermined categories. The various categories were discussed and followed with a process of reflection and discussion ending in one overarching category and three sub categories.

The researcher followed the ethical principles of doing good and respect human integrity. All 21 men were given written and oral information about the study in line with the Helsinki declaration and informed consent was obtained. Their free will to participate was emphasised and, respondents who felt their integrity violated had the right to refrain from participation. Their right to drop out of the study without any reprisals and consequences for former health care contacts was stated. In the final year of data collection, a new contact was taken with the Ethical Committee who granted the extended part of the study. A written invitation about the follow-up interview was mailed out, answered in writing and, mailed in. The researcher, LJ, personally, by phone, repeated the study information and booked time for interview in the men’s homes or workplaces. Six men accepted further participation at this stage. Information on the patients’ initial medical treatment was retrieved from medical records. The study was approved by the Ethical Committee, the Medical Faculty at Lund University, Sweden (U451-01).

## Results

Thirty-six men were consecutively asked to participate in the longitudinal study. Twenty-one men accepted, forming a convenient sample [21]. The men whom declined participation stated age, no time or no interest as reasons. Men participating were between 53 and 85 years old, 18 were married and three were widowers or lived alone. Most men had a nine-year education level, *i.e.* elementary school level, and 11 men were still working. At time of the first data collection it was between 1 and 2 years since the treatment started. Gleason scores were between 3 to 9 and PSA levels between 0.5 to 13.2 at time of diagnosis. Eleven men had had a radical prostatectomy, 6 hormonal treatments, and 4 men had had external beam radiation. There was four drop outs from the study over the three years.

### Quantitative ratings (n = 21)

The ratings for the HLQLQ C-30 (Table 1) showed high scores in both physical and role functioning, representing few physical restrictions and few restrictions in performing overall daily life activities. The degree of high functioning remained over the three years. Somewhat lower scores were found for the emotional, cognitive and social functioning. Scores did not significantly increase over the three years.

**Table 1:** EORTC HLQLQ C-30 and PR-25 module ratings over 3 years, m (sd) (n = 21)

**Year/Test**	** 1**	** 2**	**Wilcoxon**^**1)**^	** 3**	**Friedman**^**2)**^
Physical functioning	94.8 (7.4)	90.0 (19.7)		92.1 (13.8)	
Role functioning	93.3 (13.7)	91.2 (24.4)		91.7 (23.6)	
Emotional functioning	80.2 (16.8)	77.2 (20.8)		77.5 (22.0)	
Cognitive functioning	84.9 (15.7)	80.7 (23.7)		83.3 (23.6)	
Social functioning	81.1 (23.5)	80.0 (21.9)		78.4 (21.9)	
Global Health/QLQ	74.6 (23.6)	82.0 (17.2)	<0.001	81.9 (21.9)	<0.001
Fatigue	17.4 (23.2)	21.6 (24.7)		20.9 (17.7)	
Pain	7.5 (18.7)	8.9 (19.5)		6.9 (14.5)	
Nausea/vomiting	3.9 (8.9)	2.6 (8.4)		3.9 (7.3)	
Dyspnoea	9.5 (15.4)	17.5 (20.4)	<0.03	19.6 (23.7)	<0.015
Appetite loss	7.9 (20.8)	12.3 (31.8)		9.8 (25.7)	
Insomnia	16.6 (20.2)	20.4 (28.3)		17.6 (26.7)	
Constipation	14.3 (27.0)	7.0 (17.8)		5.8 (13.1)	
Diarrhoea	3.2 (10.0)	7.0 (14.0)		7.8 (14.6)	
Financial difficulties	-	10.5 (27.3)		5.8 (13.1)	
Sexual activity	27.8 (23.1)	33.3 (29.7)		28.4 (20.2)	
Sexual functioning	71.7 (29.1)	61.5 (21.3)		38.9 (20.5)	<0.008
Urinary symptoms	28.3 (29.2)	21.9 (22.5)		20.2 (16.6)	<0.05
Bowel symptoms	11.3 (14.1)	11.7 (16.3)		12.8 (18.8)	
Hormon treatm. related symptoms	17.1 (16.1)	21.2 (13.3)		17.8 (10.1)	
Incontinence aid	33.3 (−)	55.6 (38.5)		11.1 (27.2)	

^1)^Comparisons between 1st and 2nd year, (2 drop outs), ^2)^Comparisons between three years (2 drop outs).

For HLQOL scale ratings of fatigue, pain and nausea and vomiting, the sample reported low scores representing a low level of symptomatology or problems. There was a tendency to report higher levels of fatigue over the years. The difference did not exceed a 10-point scale difference and was therefore not clinically interesting. There was an increase in dyspnoea between the first two years (p = <0.03) and the three year comparison (p7 = <0.015). Low levels of financial difficulties were reported, and there was no change over the years. The overall health and life quality scale was reported low at first rating, 74.6, with an significant increase for years two (p = <0.001) and three to 81.9 (p = <0.001).

Regarding sexual life aspects (Table 1), the sexual functioning was rated lower and lower over the years with a significant lowered function (p = <0.008) in year three. Sexual activity ratings were already low at first year with no significant difference over the years. Urinary symptoms got better and problems had significantly decreased in year three (p = <0.05). Bowel symptoms and hormonal treatment related symptoms did not significantly decrease over the years. Only 1 to 6 men reported use of continence aid and the figures in Table 1 are to be interpreted accordingly.

Mean ratings for Sense of Coherence ranged between 76.2 and 74.2 (n = 21), for the study representing a medium to high SOC. With no difference in ratings between the three years (Table 2). There was a statistical correlation between cognitive functioning and quality of life over the three years and between cognitive functioning and fatigue from year two to three (not in table). There are no stipulated normal values, and Antonovsky recommended that high and low scores be set with the use of quartiles or other grouping methods for each sample. In the study, sample quartiles are used for determining low and high SOC [22]. One third of the respondents with low scores (≤ 67) were included in Low SOC and one third of the respondents with high scores (≥ 83) were included in High SOC. There was a significant correlation in years 2 and 3 between high SOC scores and general quality of life scores. Over the three years, there were significant correlations between high SOC scores and cognitive functioning and fatigue. Alpha values [23] assessing internal validity showed scores from 0.82 to 0.88 over the three years.

**Table 2:** **Sense of coherence ratings over three years (m) respondents (n = 21**)

**Year**	**1**	**2**	**Wilcoxon signed rank test**^**1]**^	**3**	**Friedman ANOVA**^**2)**^
Mean	76.2	76.1	p 0.75	74.2	p 0.68
Quartiles					
25	68.5	66.0		60.0	
50	79.0	78.0		79.0	
75	85.5	83.0		85.0	
α	0.85	0.82		0.88	

^1)^ Comparisons between 1st and 2nd year.

^2)^ Comparisons between all three years.

### Qualitative analysis complementing quantitative ratings (n = 6)

Six men accepted participation in the qualitative interview. The men were between 64 to 72 years old, some with no increase in prostate cancer illness, some with complementary hormonal and/or radiation treatment. Initial Gleason scores were between 2 to 6, and PSA levels between 4.3 to 8.7. All men were still married living with their spouse. Four men had an elementary school level education, two had higher. Four men were retired and two were still in employment. At time of interview no biological/chemical data was gathered. Men whom declined further participation were stating age, loss of energy and increased illness as reasons for non-participation. Mean ratings for SOC was between 80.0 to 78.8 (ns). Qualitative findings from semi structured interviews revealed one overarching category, Having reclaimed daily life and, three sub categories, Having vivid memories from diagnosis message occasion, Having a unique illness history, including one new highlighted aspect and, Having accepted physical shortcomings. Thus, the most prominent interpretation from the interviews was that after several years of illness and treatment the respondents were now back to daily life at a manageable and meaningful, yet different level. The result is mirrored in the sense of coherence rating which showed to be stable over the years.

### Having reclaimed daily life

Life was experienced to be good and the period of illness had given an insight into how important it was to stay ‘healthy’ and to appreciate life. In quantitative ratings overall quality of life was increasing significantly over the years underpinning qualitative results. Physical functioning such as doing strenuous tasks and managing ones dressing and hygiene were rated high complementing interview findings.

*I am fine, I think. I feel good. You feel, you feel you are not the same person as you were before the diagnosis. Life is basically like it was before*.

The men and their spouses had experienced a deepened relationship and they showed each other more attention and kindness than before. There was an expressed gratitude for life, even if it was now mixed with an anxiety for relapse and thoughts of death. Their stories expressed expectations for the future in descriptions of holidays to come and trips to make,

When all this (treatment) is over and done with, we are going to travel.

The result is complemented in quantitative ratings since aspects of social functioning such as a functioning family life and social activities were rated high and not affected over the years. Also aspects of cognitive functioning such as ability to concentrate and remembering things contributed to life normality, as did no economic problems.

A new highlighted aspect in the result not included in the predetermined categories was the adult children’s reactions to illness. Children took the message of their fathers’ cancer illness in different ways and each child needed to be met with individual consideration.

*It is difficult to talk to the children. Well, not for me, but for them. They ask how I feel, of course, but I understand that they have difficulties in what to say and you cannot sit down and have a proper talk*.

The men met each child’s unique reaction according to how much the child ‘could take’ and the men kept up an appearance of good mood and longevity. This put existential pressure and bewilderedness on the men. The result is complemented in low quantitative ratings of emotional functioning represented in terms of being tense, worried, irritated and being ‘low’. Emotional functioning was the functioning scale rated most affected among the various scales.

### Having accepted physical shortcomings

The first issue brought up about physical shortcomings were shortcomings in sexual activity and performance. Some said it was not that important any more, but the feeling of loss was not to be mistaken. Some of the sexual ability had come back, but the sex life was forever changed. Some men were helped with medication, *e.g.* Viagra, but not all of them. Sexual aids in other forms were said to hinder spontaneity and were rejected. In quantitative ratings sexual activity was rated low from the start of the study and over the years. Sexual functioning was rated high at the start and was significantly decreasing over the years complementing interview findings.

I have a fairly normal life, but I have given up some things in my married life. We were both aware of that. We have been married for 40 year and we will handle this (sexual life shortcoming) also.

Those who had urinary trouble after surgery and had been forced to wear diapers and catheters for a long time had strong reminiscences of not being clean and how they had struggled to follow recommendations of exercises to get their continence back as soon as possible. This is complemented in quantitative ratings in problems decreasing over the years. Ratings of urinary problems decreased in quantitative ratings and were not given much attention in qualitative aspects. Same is found in other side effects mentioned, such as in aspects of fatigue, pain, hormone treatment related symptoms (weight loss), nausea and vomiting (feeling sick), appetite loss and, bowel problems. Problems were rated to be the same over the years and were not highlighted in the qualitative findings.

### Having vivid memories from diagnosis message occasion

It was apparent how well the men remembered the diagnosis occasion. Date and even time of day were remembered.

*The 7*^*th*^*of January, and that is a date I remember. I thought, is this the way it (life) will be and, I understood the seriousness in the message and it was on the 7*^*th*^*of January.*

There was astonishment regarding the diagnosis that came ‘out of the blue’ because there had not been any signs of illness. There were also expressions of that the diagnosis was expected and that some had experienced premonitions of seriousness. The information received was good and the men were supposed to use it to make a decision on a treatment regime. It was difficult to choose between treatments and they were uncertain of which one was the right choice. Before surgery, they were well informed and when the decision was made they were relieved and no one regretted their choice. They were well informed about side effects and what consequences could be expected. They also knew how to keep track of illness progression. After surgery, the staff was considered stressed and had much to do, and the men missed chatting to nurses and physicians.

### Having a unique illness history

The men were well aware of how common prostate illnesses were and that many men were confronted with it. Some men also had relatives with prostate cancer or knew about men having it. Many stories about treatment and side effects were in circulation and they were often worst case scenario descriptions. One man spoke out his frustration over a neighbour,

*Every day when I met him (neighbour), he spoke about new obituaries in the newspaper of men with cancer. Finally, I said to him to shut up and not talk about it anymore*.

The men had their own illness stories and did not want to listen to stories of other men. This meant that they were hesitant to bring up the subject of cancer with friends and relatives. The men spoke to family members and to other people in the same situation. At the ward or in a waiting room, they were surprised to experience how easy it was to speak to strangers with the same or other urological diagnosis.

## Discussion

The result from this study is to be regarded within a mode of analytical generalization, and only occasional generalisations can be made outside the sample [21]. There are threats to internal validity with regard to sampling, because the men in the study are few and were probably the healthiest and most fit to participate in the study. They were also younger than the average man with prostate cancer. However, their ratings and stories are of equal value, as are those in a worse functional and emotional state, and contributes similarly to the building up of evidence-based knowledge regarding prostate cancer. To strengthen internal validity, two sources of evidence were used with a time interval between, self-rated quantitative data and qualitative interview data. The instruments used, HLQOL C-30, PR 25 and SOC-scales are well known and have proofed to be sound and reliable. There are only four drop outs in all over the three years.

Threats to reliability are mostly due to small sampling. The in-depths interviews were performed with a trained interviewer (LJ) and the use of the directed content analysis which added to previous research results did account for reliability and sensitivity in data collection. Larger samples with robust quantitative design have presented results in line with ours [22-24].

The result section is presented in a way that claims an ability of complementing qualitative with quantitative findings which could be questioned. Quantitative questions are not put to patients in the way they are presented in the questionnaires and when qualitative findings are presented as complements this is done as a results of the researchers. Our study show that the result from the combination of methods revealed aspects from quantitative ratings that where not all mentioned in the interviews and aspects raised in interviews were also others than in the quantitative assessment, confirming and complementing results and also verifying the chosen design.

From a quantitative perspective, the most startling result is the relatively high rating of overall health/life quality and functioning that seemed to be mildly touched by the cancer disease and treatment after three years. In a Swedish study, (n = 481), of men and women with cancer (prostate, breast, colorectal and, gastric cancer), and with various treatments, the rating for quality of life was comparable to our study in the first year of assessment. Ratings in that study did not differ for a period of 24 months [11], whereas life quality ratings in our study significantly increased over the three years. This is in line with results from other studies of cancer survivors showing high quality of life in men living with prostate cancer two years after treatment [25]. Reasonably this phenomenon relates to having regained ‘normal’ lives and the survival of cancer.

The qualitative interviews complemented the quantitative perspective and also gave a picture of a good life with high life quality. The finding is important with regard to clinical practice and how questionnaire results are considered in practice. When quantitative and qualitative results are measured parallel this gives a detailed foundation for action. A combination between quantitative and qualitative measures also minimizes the risk of mistaking patients’ opinions and highlights what needs they themselves put in the foreground.

Sexual functioning decreased over the years, not followed by decreased sexual activity, which did not. This was interpreted to show that the men still experienced their sexual functioning at relatively high level at the start of their treatment even if they reported low sexual activity and that the sexual functioning worsened over the years with a sexual activity on the same level as before. In a Swedish study (n = 80) of Wahlgren *et al.* with men ≥ 6 months after radiation therapy scorings are in comparison though with a less spread in our sample. The comparison is complicated since their descriptive statistics are presented differently from ours [26]. Our findings, from the quantitative assessment, are in line with interview finding, *i.e.* sexual life functioning had decreased and was missed, but in contrast to having survived cancer and considering other sides of life to be good, impact from shortcomings in sexual life were overlooked.

In the interviews, there was very little mentioned of problems with fatigue, pain, nausea and vomiting even if such problems are well known in prostate cancer care. We expected symptom scales and single items to show high degree of functioning and few problems considering the years that had passed since treatment started. This expectation was confirmed. The different areas of functioning were in line with interview findings, *i.e.* was scored to show a high grade of functioning, and these findings are coherent with a national stratified sample of men and women without a cancer diagnosis (n = 3 919) [20], representing a ‘normal’ amount of dyspnoea and diarrhoea. Symptom of dyspnoea increased and, is surprising. It may well be that symptoms comes from co-morbidity not assessed in the study. Over all, the men’s quantitative ratings were confirmed in the qualitative interview findings.

Sense of Coherence scores were medium to high for the sample and consistent over the three year. There was also a statistical correlation between cognitive functioning and quality of life over the three years and between cognitive functioning and fatigue from year two to three. This is in line with ratings from earlier studies [12]. Our results confirm that high SOC is a determinant for a perception of good health and generally high quality of life in that *e.g.* all functioning rates and life quality were high in the sample. We also suggest that the high SOC is confirmed in the interview interpretation stating that the men said to be in good ‘health’ and to be handling their life with prostate cancer sequels. For the interview findings of high sense of coherence, they support earlier research results from, among others, Nilsson *et al.*, who found a high SOC score related to high perceived health, social and emotional support [5].

To limit the influence of bias, Hsieh and Shannon suggest the use of a theory to guide the discussion of the findings. Therefore, we chose to apply the shifting perspectives model by Paterson [27], on qualitative findings. The model is building on the assumption that living with a chronic illness is an on-going process without end, in which people experience an ever-changing perspective of their illness, which makes it possible for them to make sense of their experiences. Perspectives of illness contain both illness and wellness and people put the former or latter in the foreground at different times of the illness period. Perspectives of illness also determine how people act and respond to their next of kin, caregivers, and different situations. It is the perspective of illness, not the illness it selves, that forms the way people behave, showing a behaviour that may be irrational in the eyes of others. The men, who had survived prostate cancer, may be said to live with a chronic illness, even though they are medically declared healthy, in that they explained how they lived with a constant risk of relapse. When analyzing the results in relation to the shifting perspectives model, the results make sense. It seemed to be natural for the men to state some presence of physical problems and at the same time state good health with high life quality. This is underpinned by Paterson, who argues that patients’ perspectives may be shifting over time and between different situations.

In the study, the men were putting illness in the foreground close to surgery, when still in hospital, and the first weeks at home. Their stories tell of physical and existential issues that they wanted to talk about to the staff. However, the staff was busy and had little time for patients’ problems. The importance of staff talking to patients is described by Telford *et al.*, who suggested that staff should actively listen to patients’ stories, and that they should refrain from categorizing all patients with the same illness in the same way [28].

The men remembered the diagnosis event vividly. That is not surprising, considering they were presented with a life-threatening cancer diagnosis. Little is known about what reactions are determinants for the ability of coping with a cancer diagnosis and handling its consequences in men with prostate cancer. Halbert *et al.* found that even though men are making an active effort to suppress their thoughts about cancer, they are also trying to make sense of their diagnosis. The authors found that married men and men with a high educational level seemed to handle the message of cancer better than those living alone and with low educational level [29]. In our study, most men were married, however with limited educational background. Having a spouse was an important source of support, not only at the time of diagnosis but throughout the rest of their lives and, may have contributed to sense making. The men’s stories highlighted the importance for staff to create the diagnosis events into a friendly and humanitarian atmosphere. There should be no time limits for the event and the physician and nurse attending should be well atoned to the patients’ reactions and questions.

Breaking the news of cancer to adult children was a difficult task that made a deep impression on the men. The emphasis on this aspect is a new finding revealed in this study and not recognized in our previous work. When confronted with their children’s sadness, they felt an extra existential burden on themselves. This also gave grounds for thoughts of death and the end of life. When at home, the men were striving to come back to their former life and, relied on spouses and children for communication and physical help, if needed. To get help was complicated by the fact that they did not want to put extra burden on the children.

The men avoided telling their story to people who countered with stories of their own that were often terrible stories with lethal outcome. Talking to fellow patients seemed natural, and gave some comfort. After the first months with physical shortcomings and surgical aftermaths, they slowly found their way back to ‘normality’. Willener and Hantikainen found (n = 11) that men were satisfied with their lives already after three to four months after prostatectomy, even if they were impotent and some of them had to wear diapers at the time of interview [30]. This is in line with our earlier statement that a knowledge of a successful curative treatment gives hope of survival and do not mean the end of life.

## Conclusions

The aim of this study was to describe men’s experiences of life following prostate cancer from health-related quality of life and a with a salutogenic perspective and, in an extended part of the study, complement quantitative data with qualitative descriptions. Our results show that the men’s life situation was interpreted to be good. Over the years, a shifting perspective to wellness was the dominant perspective, because the men said they had regained a good life, yet in a new way. The men’s sense of coherence seamed to support their handling of life three years after prostate cancer treatment. The interpretation is supported in the study by quantitative results showing a high degree of functioning. The men’s experiences give hope and confidence to men in the same situation and their next of kin and, to the staff caring for men with prostate cancer. Results also support the shifting perspectives model of chronic illness.

### Clinical implication

It is suggested that both qualitative and quantitative assessments are parallel performed and used as complement for each other. It is also suggested that diagnosis, treatment options and side effects from treatment are discussed with patients in a calm setting and atmosphere.

### Further research

Research is needed on comparing quantitative and qualitative assessments and on large samples to further build on a sound ground of knowledge. It might also be of interest to re-validate questionnaires phrasing since wording and phenomena undergoes changes as society and everyday language changes, *i.e.* the phenomenon of fatigue, does that mean today what it meant 20 years ago?

## Competing interests

The authors declare that they have no competing interests.

## Authors’ contributions

LJ carried out the interviews, performed the statistical tests and drafted the manuscript. LJ, LP and PL participated in the design of the study, analyzed data and, LP and PL assisted in manuscript writing. All authors read and approved the final manuscript.

## Pre-publication history

The pre-publication history for this paper can be accessed here:

http://www.biomedcentral.com/1472-6955/12/11/prepub
